# The Antitumor Effect of Topotecan Loaded Thiolated Chitosan-Dextran Nanoparticles for Intravitreal Chemotherapy: A Xenograft Retinoblastoma Model

**DOI:** 10.18502/jovr.v18i1.12727

**Published:** 2023-02-21

**Authors:** Elham Delrish, Mahmoud Jabbarvand, Fariba Ghassemi, Fahimeh Asadi Amoli, Fatemeh Atyabi, Saeed Heidari Keshel, Alireza Lashay, Farnaz Sadat Mirzazadeh Tekie, Masoud Soleimani, Rassoul Dinarvand

**Affiliations:** ^1^Translational Ophthalmology Research Centre (TORC), Farabi Eye Hospital, Tehran University of Medical Sciences, Tehran, Iran; ^2^Retina & Vitreous Service, Farabi Eye Hospital, Tehran University of Medical Sciences, Tehran, Iran; ^3^Department of pathology, Farabi Eye Hospital, Tehran University of Medical Sciences, Tehran, Iran; ^4^Nanotechnology Research Centre, Faculty of Pharmacy, Tehran University of Medical Sciences, Tehran, Iran; ^5^Department of Pharmaceutics, Faculty of Pharmacy, Tehran University of Medical Sciences, Tehran, Iran; ^6^Department of Tissue Engineering and Applied Cell Science, School of Advanced Technologies in Medicine, Shahid Beheshti University of Medical Sciences, Tehran, Iran; ^7^Department of Hematology, School of Medical Sciences, Tarbiat Modares University, Tehran, Iran

**Keywords:** Chemotherapy, Intravitreal, Nanoparticles, Ocular Malignancy, Retinoblastoma, Topotecan

## Abstract

**Purpose:**

This research intended to fabricate the thiolated chitosan-dextran nanoparticles (NPs) containing topotecan (TPH-CMD-TCS-NPs) to assess the ability of NPs in improving the efficacy of intravitreal chemotherapy of retinoblastoma in a rabbit xenograft model.

**Methods:**

The coacervation process was used to produce the NPs. The cellular uptake of Cyanine-3 (CY3)-labeled NPs were investigated in human retinoblastoma Y79 cells using confocal microscopy. Also, the prepared TPH-CMD-TCS-NPs were tested *in vitro* by the tetrazolium dyes II (XTT) and flow cytometry in order to assess their cytotoxicity. In addition, a rabbit xenograft model of retinoblastoma was developed to test the antitumor effectiveness of TPH-CMD-TCS-NPs through intravitreal administration.

**Results:**

NPs had a mean diameter, polydispersity index, and zeta potential of 30 ± 4 nm, 0.24 ± 0.03 and +10 ± 3 mV, respectively. NPs (IC50s 40.40 compared to 126.20 nM, *P* = 0.022) were more effective than free topotecan as a dose-based feature. The tumor reaction to intravitreal chemotherapy with NPs was measured by evaluating the percentage of necrosis in the tumor tissue (91 ± 2%) and vitreous seeds (89 ± 9%) through hematoxylin and eosin (H&E) staining. In comparison with the control group, the TPH-CMD-TCs-NPs treated group showed a significant decrease in tumor volume seven days after the intravitreal injection (*P* = 0.039). No significant changes were found in the ERG parameters after the intravitreal injection of TPH-CMD-TCs-NPs or TPH (*P* > 0.05).

**Conclusion:**

This investigation revealed definitive antitumor efficacy of TPH-CMD-TCS-NPs by intravitreal administration in the rabbit xenograft retinoblastoma model.

##  INTRODUCTION

For several decades, external beam radiation (EBR) was the only possible way to preserve an eye with retinoblastoma (Rb) vitreous seeding. Intravenous chemotherapy (IVC) was an approach used for treating Rb without the increased rate of secondary malignancies related to the EBR procedure. However, at least half of the eyes with vitreous seeding treated with IVC were eventually enucleated.^[1,2]^ In recent decades, intra-arterial chemotherapy (IAC) which uses a maximal local concentration of chemotherapy agents by direct application to the tumor site has significantly improved the management of Rb.^[3,4,5,6,7,8]^ Although, most eyes with vitreous seeding can be saved with this method, vitreous seeding still remains the main drawback leading to enucleation in IAC-treated eyes. Therefore, the use of intravitreal chemotherapy (IVitC) has been selected as an option to increase the globe salvage rate.^[9,10,11,12]^ Although a few chemotherapy molecules have been effective in the treatment of Rb, these medications have not achieved the optimal concentrations needed within the ocular malignant tissue. Insufficient delivery of these small molecular drugs causes dose-limiting side effects including toxicity and limited bioavailability. For more efficient drug delivery and targeting of Rb, nanotechnology may be an implementable strategy. Nano-carrier material has been shown to be a successful drug delivery system (DDS).

Applying non-degradable drug carrier materials can give rise to an additional problem of proper elimination of such particles from the vitreous after release of the medication. Chitosan (Cs) is a biocompatible, biodegradable, and non-toxic linear polysaccharide. Since it has free –OH and –NH ${}_{2}$ groups in its construction, it is also amenable to chemical modifications for potentiating some of its features for particular purposes^[13,14]^ Trimethyl chitosan (TMC) is a water-soluble derivative of Cs fabricated by N-methylation of some of Cs's free amine groups using iodomethane.^[15,16]^ Centered on the immobilization of the thiol group on the TMC backbone, thiolated chitosan (TCs) is synthesized, leading to improved biopolymer mucoadhesive properties.^[17]^ Carboxymethyl dextran (CMD) is a hydrophilic polymer made of many glucose molecules, often consisting of α-1, 6-glycosidic bonds,^[18]^ which is used to enhance the bioavailability of anticancer drugs in malignant tissue. High degrees of methylation (DQ%) in TMC-cysteine conjugates (TCs) used in this study might have led to high rates of O-methylation that resulted in less solubility of TCs-NPs. Therefore, using CMD improves stability and impedes O-methylation. It also contributes to NP's reactivity owing to its carboxyl and hydroxyl groups.^[19,20]^

Topotecan hydrochloride (TPH) is approved for the prevention of cervical cancer, breast cancer, and small cell lung cancer.^[21,22,23]^ Additionally, TPH is also identified as a potent drug against Rb with a safe toxicity profile.^[24,25,26,27]^ TPH has been used as a systemic chemotherapy for resistant intraocular Rb.^[28]^ Periocular injection of topotecan has been handled in patients with recurrent tumors and in those who are not inclined to systemic chemotherapy.^[29]^ However, efficiency limitations have shown that TPH's active lactone form is vulnerable to pH-dependent hydrolysis of the biologically inactive carboxylate species under physiological conditions, which presents a barrier to its therapeutic efficacy.^[28,29]^ By encapsulating topotecan in CMD-TCs-NPs, the stability concern of the topotecan might be solved. In addition, the bioavailability of TPH in malignant tissues may be improved due to CMD-based surface stabilization, which decreases agglomeration after the intravitreal NP injection. In the present analysis, TCs and CMD were used to create an efficient topotecan carrier to increase the bioavailability and avoid the agglomeration of intravitreal TPH-loaded NPs.

##  METHODS

Cs medium-molecular-weight Chi with a degree of deacetylation of about 89% was purchased from Primex (Karmoy, Norway). TPH was supplied by Yangzhou Huaxing Chemical Company, China. CMD sodium salt (10–20 KD, 1.1–1.5 mmol carboxyl/g), dialysis tubing (molecular weight cut-off 2 and 12 kDa), N-ethylcarbodiimide hydrochloride (EDC), N-hydroxysuccinimide (NHS), Ellman's reagent, RPMI-1640 tissue culture medium, and fetal bovine serum (FBS) were purchased from Sigma-Aldrich Company (Missouri, USA). N-methyl-2-pyrrolidone (NMP) and hydrochloric acid (HCl), sodium chloride, and sodium hydroxide (NaOH) were purchased from Merck Company (Darmstadt, Germany). The human Rb cell line (Y79) was obtained from Pasteur Institute (Tehran, Iran) and cell-counting solution (Orangu TM) was prepared by Cambridge Bioscience. All chemicals were of analytical grade.

### Synthesis and Characterization of TMC

The synthesized TMC followed the process defined by Sieval et al.^[30]^A suspension of Cs (2 gr) was prepared in NMP (80 ml) and was immersed for 5 min – a short period of time. Eleven ml of 15% NaOH and 11.5 ml of methyl iodide and 4.8 gr of NaI were applied to this solution, followed by a reflux at 60ºC for 2 hr. Then, 2.5 ml of methyl iodide and 0.7 gr pellets of NaOH were added and stirred for 1 hr and precipitated by adding 200 ml of ethanol. The derivative was centrifuged, washed, and dried to obtain trimethyl Cs iodide which was dissolved in 40 ml solution of 10% NaCl to interchange iodide with chloride. Afterward, the solution was precipitated with ethanol again and the mixture was centrifuged, washed, and dried. The degree of quaternization (%DQ) of TMC was calculated using^1^H NMR spectrum recorded by a 600 MHz spectrometer (Bruker-Biospin, Germany). Data analysis was performed using the Topspin software. The %DQ was calculated by the following formula: DQ = [[(CH3)3 / [H] × 1/9] × 100.

DQ is the degree of quaternization; (CH3)3 is the integral of chemical shift of the hydrogens of trimethyl amino groups at 3.4 ppm; H is the integral of H-1 peaks between 4.7 and 5.7 ppm.^[31]^

### Synthesis and Characterization of TMC-cysteine Conjugates (TMC-cys)

The method of synthesis was adapted according to Margit et al.^[32]^ In the first step, 200 mgr of prepared TMC was dissolved in 10 ml of distilled water (DI) and then 400 mgr of cysteine was poured and stirred until dissolved. Then, EDC and NHS were added in a final concentration of 200 mM, and the mixture was kept at room temperature and pH was adjusted to 5. Then, the solution was incubated for 3 hr in the dark under constant stirring at room temperature. Afterward, the solution was dialyzed (membrane dialysis MW cut-off = 2 kDa) using 1 mM HCl for three days at 4ºC and then dialyzed again for two times utilizing the same medium but containing 1% NaCl. Finally, the mixture was lyophilized and the conjugates kept at 4ºC. Determination of free thiol groups immobilized on the polymer backbone was performed via photometry with Ellman's reagent while the quantitative amount of the thiol groups was determined using the thioglycolic acid standards curve.^[32,33]^ FT-IR spectra of TCs were recorded on a Bruker FTIR spectrophotometer (Vectore 22, Germany).

### Preparation of CMD-TCs Nanoparticles (NPs)

The nanoparticles (NPs) were made by a simple coacervation technique where CMD was used as the cross-linking agent.^[34]^ The NPs were prepared by adding CMD solutions to TCs solutions which contain TPH. Then, an immediate vortex stirring was performed and samples were incubated at room temperature for 2 hr. The formed NPs were then washed by DI through an AmiconⓇ filter with a cut of 30 nm to eliminate untrapped TPH.

### NP Characteristics

The particle size and polydispersity index (PDI) of NPs were determined using dynamic light scattering (DLS) on a Malvern Zetasizer Nano-ZS (Worcestershire, United Kingdom). Surface charges of the NPs were measured by laser Doppler anemometry using a Zetasizer Nano Series (Malvern Instruments). The morphology of NPs was distinguished by field emission scanning electron microscopy (FESEM; ZEISS, EVo 18). To prepare samples for the FESEM study, 100 µl of the nano-particle suspension was placed on a glass, dried, and then coated with gold layer for 30 s. NPs were also stained by phosphotungstic acid (2%, w/v) and observed by transmission electron microscopy (TEM, Zeiss, EM 900) to reveal the particle size and morphology. The Fourier-Transformation Infrared (FT-IR) spectroscopy analysis was performed on a Bruker FTIR Fourier transform spectrophotometer (Vectore 22, Germany). The scanning range was from 4000 to 400 cm ${}^{-1}$ . X-ray diffraction analysis was used to distinguish the crystallinity of the pure TPH and TPH in NP-based drug formulations, which was conducted using a StoeStidy-mp x-ray diffractometer (XRD). Differential scanning calorimetry (DSC; DSC823e, Mettler) was utilized to determine the physical state of TPH in NPs. Approximately 5 mg of the sample was measured, placed into an aluminum pan, and analyzed at a scanning temperature of 24–400ºC at a heating rate of 10ºC/min. The entrapment efficiency (EE) of TPH was determined by a centrifugation method. The drug-loaded NPs were cold centrifuged at 4ºC for a period of 30 min at 3000 rpm via AmiconⓇ ultra centrifugal filter (Millipore 30kDa).^[35]^ The supernatant liquid was collected to ascertain the non-bound drug concentration by using ultraviolet-visible spectrophotometry (UV-vis)^[36]^ (Aquarius, CE 7500) at λmax 275 nm. The %EE and drug loading (%DL) were computed using the following formulas: 


%EE=weightofdrugincorporatedinnanoparticles/weightofdrugfedinitially*100.

$$\%DL=\frac{weightofdrugincorporatedinnanoparticles}{weightofnanoparticle}*100.$$

Cumulative release experiments were operated in BSS (balanced salt solution) medium (pH =7.5) to verify the amount of TPH released from CMD-TCs-NPs. NPs were dispersed into 10 ml of BSS, then incubated at 37ºC under 150 rpm. At determined time intervals, a 500 µl of the medium was withdrawn, centrifuged at 12000 rpm for 20 min to separate NPs from supernatant, and then replaced with an equal amount of fresh media to maintain sink conditions. The supernatant was used for UV examination at 275 nm (λ max).

### Evaluation of IC ${}_{50}$

The cancerous Y79 human Rb cells were obtained from the Pasteur Institute and used in this research to elucidate cell viabilities of TPH-loaded CMD-TCs-NPs. Cells were seeded at a concentration of 5 × 10^3^ cells/well on a 96-well plate and maintained at 37ºC in a humidified, 5% CO ${}_{2}$ atmosphere. After overnight cultivation, cells were treated with TPH-loaded CMD-TCs-NPs, CMD-TCs-NPs, and free TPH with equivalent TPH concentrations of 5, 10, 20, 50, 100, and 200nM, respectively, for 24 hr at 37ºC. Cell counting solution (2,3-bis-(2-methoxy- 4-nitro-5-sulfophenyl)-2H-tetrazolium-5-carboxanilide, Orangu ${}^{\text{TM}}$ , Bioscience) was used to determine the cell viability per standard protocol suggested by the manufacturer. The cells viability of Y79 cells was defined as: 


$$\frac{{OD}_{NPstreated}-{OD}_{Blank}}{{OD}_{Control}-{OD}_{Blank}}\times 100,$$

where, OD NPs treated, OD blank, and OD control represent the optical densities of treated, blank, and control samples, respectively [Table 1].

### Flow Cytometry Measurement

Apoptosis analysis using flow cytometry was used to quantitatively confirm the IC ${}_{50}$ determined by XTT assay. Y79 cells were seeded at a concentration of 2.5 × 10^5^ cells/well on a six-well plate and when reaching 70% confluence treated with 50 µM concentration of TPH-CMD-TCs-NPs and TPH for 48 hr. The extent of apoptosis was determined by Annexin-V-FITC staining using Annexin-V-Phosphatidyl serine apoptosis detection kit (IQ Products; Netherlands) as directed by the manufacturer.

### In vitro Cellular Uptake of NPs

Qualitative cellular uptake of TPH-loaded CMD-TCs-NPs was investigated with a confocal laser-scanning microscope (Nikon, Eclipse). For this, the Y79 cells were cultured in a six-well configuration at the density of 2.5 × 105 cells/well. The cells were then incubated for 2 hr with Cyanine-3 (CY3)-labeled TPH-CMD-TCs-NPs suspension medium at concentrations of 200 μg/ml to follow the uptake of them in Y79 cells. Afterward, the cells were fixed with 2% paraformaldehyde and cell nuclei were stained with DAPI (4 ' ,6-diamidino-2-phenylindole) and endosomes/lysosomes were stained with LysoTracker Red, respectively. The fluorescence of the CY3-labeled TPH-CMD-TCs-NPs was monitored applying a confocal microscope (excitation 640.8 nm/emission 662–737 nm).

### Rabbit Xenograft Model of Rb

All animals in this survey were used according to the Association for Research in Vision and Ophthalmology Statement in a protocol approved by Tehran University of Medical Sciences. Fifteen male New Zealand albino rabbits with a mean initial weight around 1 kg purchased from Pasteur Institute of Iran (Karaj, Iran) were used for this study. The rabbits were immunosuppressed with daily intramuscular injections of cyclosporin A (CsA; Sandimmune 50 mg/mL; Novartis Pharmaceuticals, Germany). Animal study groups were conducted with a sample size of *n* = 5, except for the tumor control group (*n* = 2). To avoid spontaneous tumor regression, CsA administration had been continued during the 10-week study period. All injections were performed by the same surgeon (FG). The dosage schedule was 15 mg/kg per day for 5 days before cell inoculation and followed by 10 mg/kg per day for the next 10 weeks of the investigation.^[37]^ During the 10-week follow-up, the animals were monitored daily for signs of CsA toxicity (weight loss, gingival hyperplasia, and diarrhea). Fifty µl of sterile PBS (phosphate buffered saline; Gibco, Germany) containing 2.5 × 10^6^ Y79 cancerous human Rb cells was injected intravitreally using a 30-gauge needle. At week eight, after intraocular tumor inoculation, the rabbits were anesthetized, examined, and ultrasound examinations were performed on the eyes using 10-MHz B-scan (Ultrasonix Medical; TOUCH ultrasound system, Canada, Richmond). Then, TPH-CMD-TCs-NPs (100 µg/ml) were injected intravitreally to Rb eyes (100 µL). Control eyes received intravitreal topotecan at the same concentration (10 µg) (positive control) and the tumor group (negative control) received 100 µL of saline solution. The tumor size was estimated with indirect ophthalmoscopy. The length, width, and height of the mass were all measured both clinically using a lens magnifier and ultrasonically. The results obtained from both methods were in good agreement in determining the length, width, and height. The results reported in Table 2 are related to the results of measuring the dimensions by an ultrasonic system. To estimate the mass volume, we used the approximate formula of tumor length * tumor width * tumor height. Dark-adapted bright flash ERG was done on all rabbits before the intravitreal injection at baseline and then seven days after the intravitreal injection of TPH or TPH-CMD-TCs-NPs prior to sacrificing the rabbits. All surviving animals were euthanized seven days after treatments. Enucleation was performed and paraffin-embedded tissue was cut, stained with hematoxylin and eosin (H&E), and immunostained with Bcl-2 antibody. Vitreous seeds cytologic analysis by H&E staining was also performed. Slides were quantified by an experienced pathologist (FAA) to assess the percentage of tumor necrosis and immunohistochemistry.

### Statistical Analyses

To describe the data, we used mean and standard deviation. In order to evaluate the changes within groups, we used Wilcoxon signed-rank test. Comparison between groups was performed using the Kruskall–Wallis test. In addition, multiple comparisons were performed using the Bonferroni method. We used the Kruskal–Wallis test to compare the groups within different treatment groups. In addition, any statistically significant test was followed by the Bonferroni post hoc test. In order to estimate IC50, we used linear regression analysis within each treatment group. All statistical analyses were performed by SPSS software (IBM Corp. Version 25.0. Armonk, NY: IBM Corp.). *P*-value < 0.05 was considered statistically significant. All other experiments were done in triplicate.

**Figure 1:**
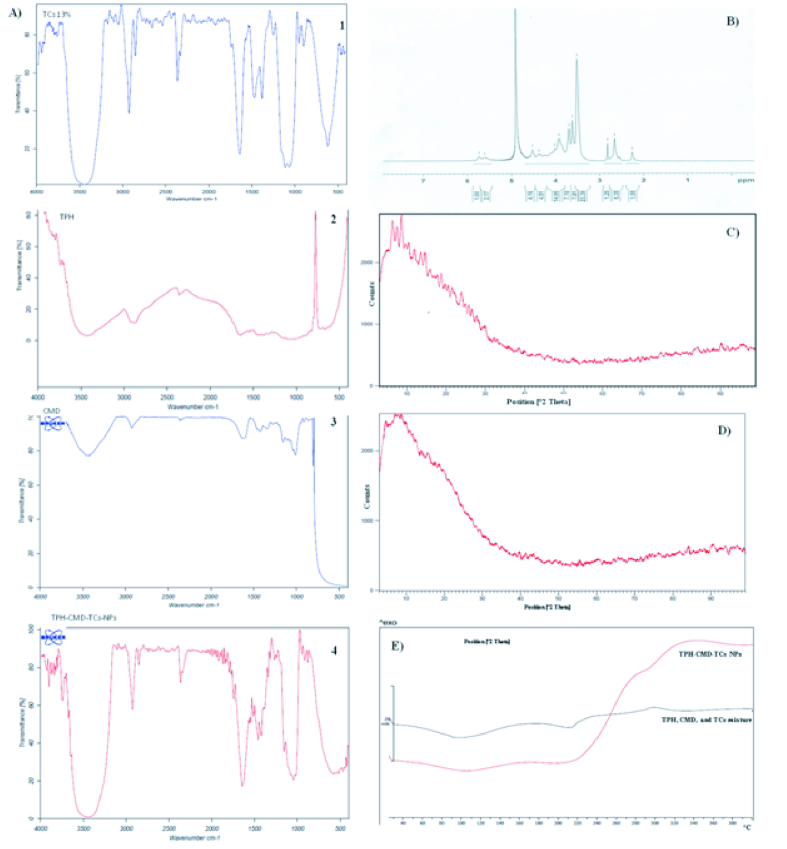
Characteristics of TPH-CMD-TCs-NPs. (A) FT-IR spectra of TCs 10% (1), TPH (2), CMD(3), and TPH-CMD-TCS-NPs (4). (B) ^1^H-NMR spectrum of TMC-cysteine conjugates in D2O. (C) X-ray diffraction pattern of TPH. (D) X-ray diffraction pattern of TPH-CMD-TCs-NPs. (E) Differential scanning calorimetric of physical mixture of TPH, CMD, and TCs, and TPH-CMD-TCs-NPs.

**Figure 2:**
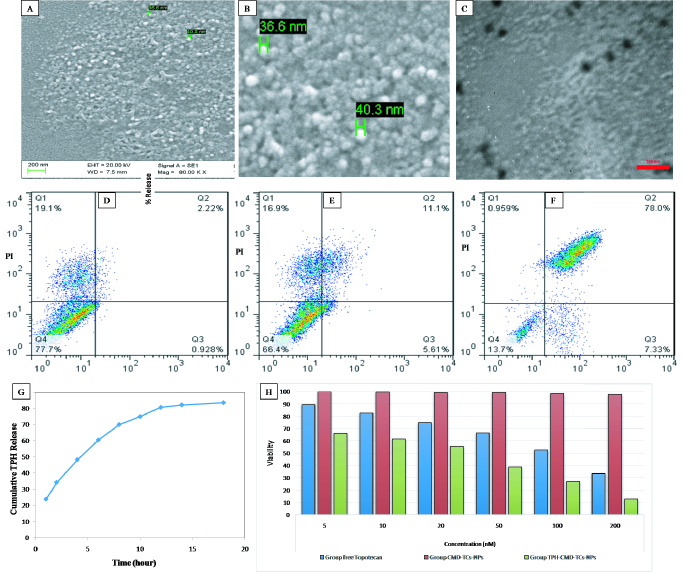
SEM images of TPH-CMD-TMC-Nps (A & B); TEM images of TPH-CMD-TMC-Nps (C); analysis of apoptosis by flow cytometry in Y79 cells (D–F). Cells were treated with 50 µM of TPH (E) and TPH-CMD-TMC-Nps (F) for 48 hr. Cumulative TPH-CMD-TCs-NPs release behaviors in BSS medium at 37ºC (G). Determination of the IC50 value of the TPH, CMD-TCs-NPs, and TPH-CMD-TCs-NPs in retinoblastoma cell line (Y79) by XTT Cell Viability Assay (H).

**Figure 3:**
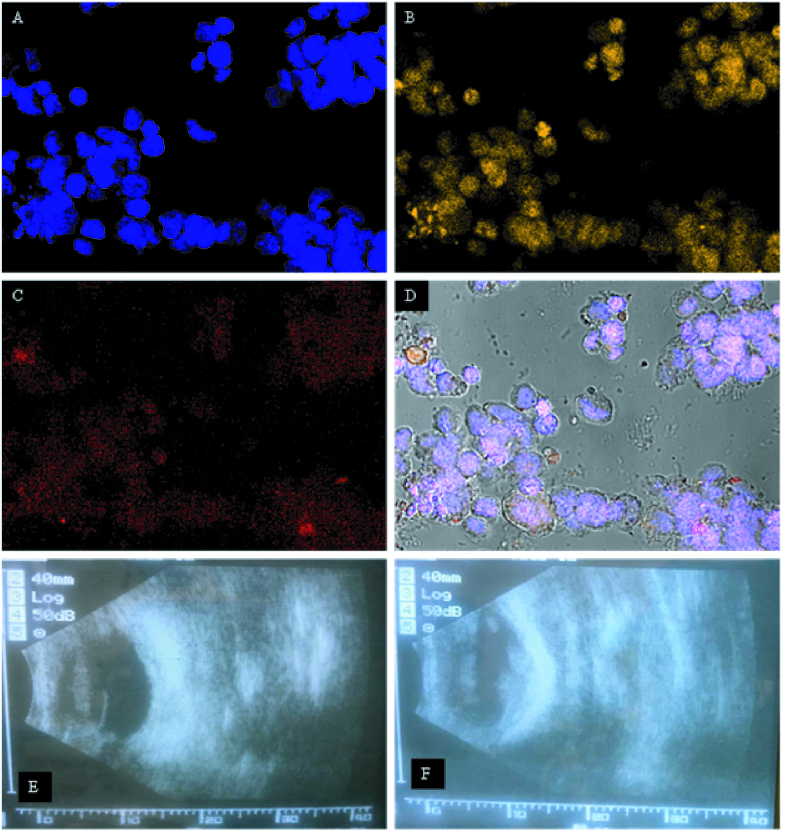
Intracellular localization of TPH-CMD-TCs-NPs in Y79 cells using CY3-labeled NPs (B) and lysosomes labeled with LysoTracker Red dye (C) observed by confocal laser scanning microscopy (A–D). Lysosomes and CY3-labeled NPs appear in red and yellow in the confocal microscopy fluorescence images, respectively. Normal Y79 cells used as control (untreated Y79 cells) (A) and nuclei stained by DAPI. Colocalization of TPH-CMD-TCs-NPs and Lysosomes in Y79 cells (D). Ultrasound image at post-injection eight weeks. The B-scan image shows an intraocular tumor in a rabbit eye (E, F).

**Figure 4:**
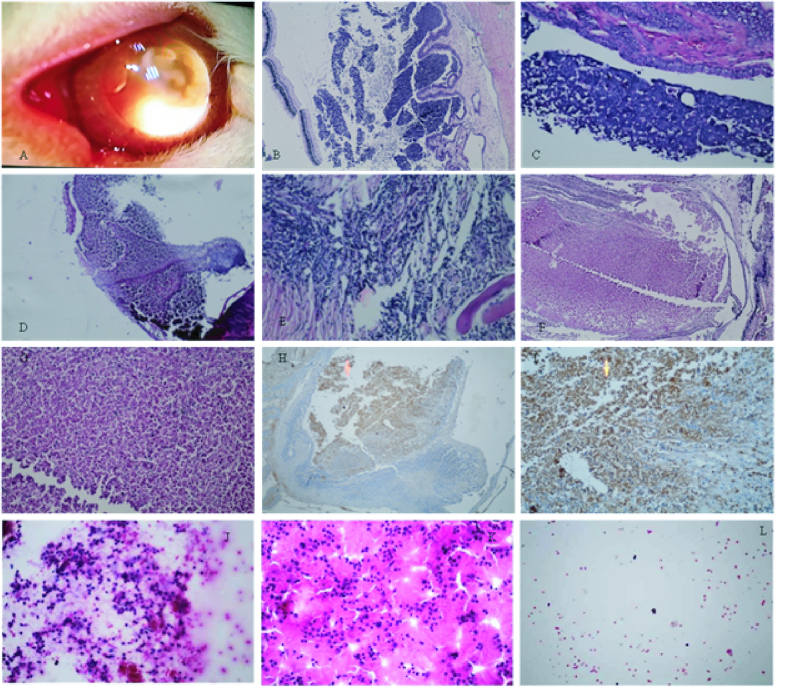
Macroscopic view of a retinoblastoma-induced eye where the tumors are seen (A). Histological findings at post-injection 10 weeks (hematoxylin-eosin) (B–F). Section from tumor control (untreated) (B & C). Section from TPH-treated tumor (D, E). Section from TPH-CMD-TCs-NPs-treated tumor (G, F). Representative light micrographs of IHC staining for BCL2 sections from TPH-CMD-TCs-NPs-treated tumor (H, I). Pathological results after the treatment of RB-induced eye with intravitreal injection of saline (J), TPH (K), and TPH-CMD-TCs-NPs (L). The ICC results showed that there was a highly significant difference in the percentage of necrosis in vitreous seeds between the NPs-treated group (89 ± 9%) and the control group (10%) (*P* = 0.046).

##  RESULTS 

### Characterization of TPH-CMD-TCs NPs

The proton nuclear magnetic resonance (^1^H NMR) spectrum of TMC-cysteine conjugates (TCs) is displayed in Figure 1B. In the TMC ^1^H NMR spectrum [Figure S1], the signal at 3.4–3.6 ppm corresponds to the methyl group at the N,N,N-trimethylated site.^[38]^The TMC-cys conjugate was synthesized based on the amide bond formation between the amino group of Cs and the carboxylic of cysteine. Comparative FT-IR spectra of native Cs and TCs are shown in Figure S2. TCs shows the three characteristic peaks at 1250, 1640, and 2500 cm ${}^{-1}$ which correspond to C–SH stretching, C = O double bonds of the amido bond, and –SH stretching, respectively [Figure 1A-1 and Figure S2].^[39]^ In addition, the degree of thiol substitution was determined at 11% using Ellman's protocol. The coacervation technique was used to obtain the NPs' suspensions. The EE and drug loading of NPs were found to be 62.41 ± 3 and 10.23 ± 0.03 %, respectively. Additionally, TPH-loaded CMD-TCs-NPs exhibited diameters of 30 ± 4 (PDI: 0.24 ± 0.03) and zeta potentials of 10 ± 3 (mV) while using DLS. As revealed by the SEM images [Figures 2A & 2B] and TEM image [Figures 2C], TPH-CMD-TCs-NPs were spherical with a compact structure. IR spectrum of TCs, TPH, CMD, and TPH-CMD-TCs-NPs exhibits their functional groups as shown in Figure 1A-1–4. The obtained peaks disclosed that there was no remarkable conversion in the polymers and drug structure in NPs and their structural integrity was maintained [Figure 1A-4]. XRD pattern of the TPH and TPH-CMD-TCs-NPs are exhibited in Figures 1C and 1D, respectively. The presence of partly sharp peaks in the diffractogram of pure TPH suggested its crystalline nature being shown, while in the case of TPH-loaded NPs there were no sharp peaks, suggesting its amorphous nature. It was clear that a broad peak was presented in the TPH-loaded CMD-TCs-NPs, indicating that NPs were amorphous and lacked crystalline peaks. Compared to the pure TPH that was represented by broad peaks, a reduction in the peak intensity could be explained by a lower loading.

Figure 1E shows DSC thermo grams of a physical mixture of TPH, TCs, and CMD, and TPH-CMD-TCS NPs, respectively. As shown in Figure 1E, topotecan in the physical mixture has a melting point of 210.66ºC, and after its encapsulation into the biopolymeric NPs, this peak has disappeared, showing that TPH is in a totally amorphous form. Results of DSC and XRD indicated that in the prepared NPs, the drug was present in the amorphous phase and might have been homogeneously dispersed in the biopolymeric matrix. As shown in Figure 2G, TPH could be approximately 70% released from the TPH-CMD-TCs-NPs in the initial 8 hr.

### Assessment of Modified NP Cytotoxicity

After TPH encapsulation into the TPH-CMD-TCs NPs, the cytotoxicity of TPH increased remarkably. As a function of the dosage, TPH-CMD-TCs-NPs were more efficacious than free TPH, following 24 hr of treatment as determined by XTT assay (IC50s 40.40, relative to 126.28 nM, *P* = 0.022) [Table 1; Figure 1H].

**Table 1:** The cytotoxicity test was conducted on the Y79 cells exposed to TPH (1), CMD-TCs-NPs (2), and TPH-CMD-TCs-NPs (3) (*n* = 3, mean ± standard error of the mean). The statistical analysis was conducted using Kruskal–Wallis and Bonferroni post hoc test.


orange**Concentration (nM)**	orange**Viability of treatment groups (Mean ± Standard Deviation)**					orange ***P*** **-value**	orange**Multiple comparison**		
	**Free TPH (1)**		**CMD-TCs-NPs (2)**	**TPH-CMD-TCs- NPs (3)**	**1 vs 2**	**1 vs 3**	**2 vs 3**
5	89.43 ± 0.94	99.93 ± 0.13	66.19 ± 0.9	0.027	0.534	0.021	0.534
10	82.41 ± 0.88	99.72 ± 0.24	61.51 ± 1.23	0.027	0.539	0.022	0.539
20	74.83 ± 1.45	99.3 ± 0.14	55.56 ± 0.99	0.027	0.539	0.022	0.539
50	66.59 ± 1.57	99.35 ± 0.08	38.88 ± 1.85	0.027	0.539	0.022	0.539
100	52.72 ± 1.45	98.53 ± 0.53	27.08 ± 1.56	0.027	0.539	0.022	0.539
200	33.53 ± 1.88	97.72 ± 0.43	12.86 ± 1.61	0.027	0.539	0.022	0.539
	
	

### Flowcytometric Analysis 

XTT viability assay outcomes were further validated by quantitative detection with flow cytometry. To that end, cell apoptosis was evaluated to compare the cytotoxicity induced by TPH-CMD-TCs-Nps and the TPH [Figures 2D & 2F].

### Uptake of Topotecan-loaded TCs NPs By Y79 Cells

The cellular uptake of Cy3-labeled NPs by Y79 cells was visualized using confocal microscope after a 2-hr exposure (3 A-D). For observation of the internalized TPH-CMD-TCs-NPs location, the nucleus was stained using DAPI (blue fluorescence), while the lysosomes were stained with LysoTracker red.

### In vivo Tumor Inhibition Study in the Rabbit Xenograft Model of Rb

Animals developed an Rb eight weeks post-injection [Figure 3E, 3F, & 4A]. The histological analysis of Rb necrosis after applying different treatments by H&E staining is shown in Figures 4B–4G. For immunohistochemistry, sections were obtained from paraffin-embedded tissue blocks and then applied for BCL2 immunostaining [Figures 4H & 4I].Vitreous seeds cytologic analysis by H&E staining detected remarkable reduction of tumor cells in seeds treated by TPH-CMD-TCs-NPs as compared with other groups [Figures 4J–4L]. In addition, Bonferroni post hoc test showed that there was a statistically significant difference in the percentages of necrotic cells in the control and NPs-treated groups *in vivo* (*P* = 0.046). Furthermore, as a function of the tumor necrosis percentage, TPH-CMD-TCs-NPs (91 ± 2%) were more efficacious than TPH (23 ± 15%). Based on Kruskall–Wallis test, there was a statistically significant difference among groups regarding the tumor necrosis value (*P* = 0.044). Wilcoxon signed-rank test revealed that the difference in tumor volume was significant in comparison with the TPH-CMD-TCs-NPs treated with tumor control group (*P* = 0.039). Safety studies were performed in rabbits to assess the potential ocular toxicity after intravitreal injections of either TPH or TPH-CMD-TCs-NPs. No significant changes were found in the ERG parameters between the non-treated (tumor control) eyes and the eyes to which either TPH-CMD-TCs-NPs or TPH was injected (*P* > 0.05) [Table 2]. In addition, as shown in Table 2, eyes showed little or no change in a- and b-waves of amplitude and implicit times values among all groups with no statistically significant difference among groups before any injection and after intravitreal TPH and TPH-CMD-TCs-NPs injections (*P* > 0.05) [Table 2].

**Table 2:** Clinical examinations and electroretinogram (ERG) before and after intravitreal injection of TPH or TPH-loaded NPs.Data are presented as Mean ± Standard Deviation (SD). Time 0 and 7 correspond to animal response before and seven days after the intravitreal injection of TPH or TPH-NPs, respectively.


orange**Parameter**	orange**Time (day)**	orange**Treatment groups**			orange ***P*** **-value † **	orange**Multiple comparison**		
	**TPH**	**TPH-CMD-TCs-NPs**	**Tumor Control**	**P1**	**P2**	**P3**
Tumor Necrosis %	7	23 ± 15	91 ± 2	15 ± 7	**0.018**	**0.044**	1	0.076
Cytologic Necrosis %	7	15 ± 6	89 ± 9	10 ± 0	**0.015**	0.06	1	**0.046**
Tumor Length (mm)	0	3.5 ± 1.3	5.8 ± 1.6	3.3 ± 3.9	0.271	–	–	–
Tumor Length (mm)	7	3.2 ± 1.2	3.2 ± 3	5 ± 4.2	0.636	–	–	–
Tumor Length (mm)	Change	0.33 ± 0.29	2.2 ± 1.3	–1.75 ± 0.35	**0.049**	0.658	0.658	**0.047**
	P ‡	0.157	0.063	0.18		
Tumor Width (mm)	0	2.8 ± 0.3	4.8 ± 1.3	2.3 ± 2.5	0.103	–	–	–
Tumor Width (mm)	7	2.3 ± 0.3	2.7 ± 2.6	4 ± 4.2	0.911	–	–	–
Tumor Width (mm)	Change	0.5 ± 0	2.1 ± 1.78	–1.75 ± 1.77	0.107	–	–	–
	P ‡	0.083	0.078	0.18		
Tumor Height (mm)	0	4.2 ± 2.5	4.8 ± 1.5	1.8 ± 1.8	0.203	–	–	–
Tumor Height (mm)	7	3.3 ± 1.9	2.3 ± 1.6	2.5 ± 2.1	0.579	–	–	–
Tumor Height (mm)	Change	0.83 ± 0.58	2.5 ± 1.17	–0.75 ± 0.35	**0.037**	0.432	0.82	**0.041**
	P ‡	0.102	**0.042**	0.18		
Tumor estimated Volume (mm)	0	49.208 ± 48.651	153.4 ± 109.439	36.063 ± 50.823	0.203	–	–	–
Tumor estimated Volume (mm)	7	29.167 ± 28.466	56.7 ± 95.487	113 ± 156.978	0.803	–	–	–
Tumor estimated Volume (mm)	Change	20.04 ± 20.2	96.7 ± 91.72	–76.94 ± 106.15	**0.033**	0.351	0.916	**0.039**
	P ‡	0.109	**0.043**	0.18		
a-wave				
Implicit time (ms)	0	15.5 ± 0.57	16.6 ± 1.13	16.07 ± 0.19	0.243	–	–	–
Implicit time (ms)	7	15.23 ± 0.18	16.25 ± 2.06	16.07 ± 0.23	0.243	–	–	–
Implicit time (ms)	Change	0.27 ± 0.38	0.35 ± 1.29	0 ± 0.42	0.92	–	–	–
	P ‡	0.317	0.715	1		
b-wave				
Implicit time (ms)	0	33.6 ± 0	35.4 ± 1.1	35.3 ± 1.1	0.119	–	–	–
Implicit time (ms)	7	34.95 ± 0.64	36.83 ± 3.05	34.23 ± 0.89	0.472	–	–	–
Implicit time (ms)	Change	–1.35 ± 0.64	–1.43 ± 2.92	1.07 ± 2.02	0.223	–	–	–
	P ‡	0.18	0.465	0.655		
a-wave				
Amplitude (mv)	0	51.83 ± 7.35	66.1 ± 7.06	66.72 ± 2.85	0.135	–	–	–
Amplitude (mv)	7	57.65 ± 5.3	63.63 ± 15.18	66.99 ± 6.2	0.57	–	–	–
Amplitude (mv)	Change	–5.82 ± 2.05	2.47 ± 8.2	–0.27 ± 9.05	0.21	–	–	–
	P ‡	0.18	0.465	0.655	–	–	–
b-wave				
Amplitude (mv)	0	83.98 ± 8.09	73.43 ± 12.1	71.3 ± 1.13	0.400	–	–	–
Amplitude (mv)	7	92.9 ± 15.7	83.67 ± 18.48	72.96 ± 7.71	0.274	–	–	–
Amplitude (mv)	Change	–8.92 ± 7.61	–10.24 ± 23.24	–1.66 ± 8.85	0.779	–	–	–
	P ‡	0.18	0.465	0.655		
	
	
white<bcol>9</ecol> ‡ Based on Wilcoxon-singed rank test; † Based on Kruskall–Wallis test; Multiple comparison based on Bonferroni method. P1, comparison of TPH and TPH-CMD-TCs-NPs groups; P2, comparison of TPH and tumor control groups; P3, comparison of TPH-CMD-TCs-NPs and tumor control groups

##  DISCUSSIONS

Nanotechnology-mediated chemotherapy has been an important development in clinical research that can enhance bioavailability and therapeutic effectiveness with negligible side effects on normal tissues. The key feature of nano-carrier innovation in this research was the development of a successful anti-cancer DDS based on biopolymers, thanks to the desirable characteristics of these nano-carriers. Chemical characteristics of Cs were employed to synthesize a water-soluble derivative that is dissolved in neutral pH. The NPs were fabricated by two hydrophilic polymers – TMC-cys (TCs) and CMD – to optimize the delivery system of topotecan for intravitreal chemotherapy. Similar to Cs, TMC also possesses muco-adhesion potential.^[40]^ It has been proven that DQ has a significant effect on the muco-adhesive properties of TMC. Snyman et al observed that the muco-adhesiveness of TMC decreased with an improvement of DQ between 22.1 and 48.8%.^[41]^ Therefore, the TMC-cys conjugate was fabricated to solve the problem and increase the muco-adhesion properties of fabricated NPs. Additionally, the solubility of TCs-NPs may decrease as a result of high rates of O-methylation in TMC-cys conjugates. Therefore, a new strategy of combinations of CMD and TMC-cys conjugates as polymeric matrices in NPs fabrication was used in this study to increase the solubility and prevent agglomeration of NPs [Figures 2C & 2G).^[19,42,43]^Accordingly, in the present study, we prepared TPH-CMD-TCs NPs by electrostatic interaction between positively charged amine groups of TCs and negatively charged CMD. The results of FTIR spectroscopy confirmed the TPH-CMD–TCs NPs formation [Figure 1A].

The optimal formulation of CY3-labeled TPH-CMD–TCs NPs showed effective cellular uptake in the Y79 cell line [Figures 3A–3D]. Therefore, by the addition of CMD to TCs, NPs with proper particle size, zeta potential, and high cellular uptake were created. Herein, we explored the possible utility of TPH-CMD-TCs to induce apoptosis in Y79 cells in order to improve the effectiveness of Rb therapy. As shown in Figure 2H, provided that apoptosis induced in Y79 cells increased significantly in the presence of TPH-CMD–TC NPs as compared to TPH, the administration of these NPs has been shown to be more efficient in the management of Rb.

We then assessed the treatment efficacy of TPH-CMD-TCs-NPs following intravitreal administration in the xenograft Rb model in rabbits. The tumor size was measured just before the intravitreal injection and seven days after the intravitreal injection of NPs. As shown in Table 2, increased and decreased tumor size over time in animals was observed in the control tumor and TPH-CMD-TCs-NPs-treated groups, respectively. As a result, apparent suppression of Rb tumor growth in NPs-treated rabbits was observed. The H&E staining findings [Figures 4B–4G] revealed that the sections of the tumor of saline-treated rabbits were densely cellular, while those of the TPH-CMD-TCs-NPs-treated population were less cellular. Light microscopic assessments revealed no histologic evidence of retinal damage induced by intravitreal administration of NPs. Also, no significant differences were found in the ERG parameters between the tumor control eyes and the eyes that received intravitreal injections of NPs (*P* > 0.05). Therefore, TPH-CMD-TCs-NPs could be considered safe for the retina.

Cs-based NPs are particularly valuable due to their low toxicity and biocompatibility. These nano-carriers demonstrate high efficacy and safety for cancer therapy both *in vitro* and *in vivo*.^[44]^ In this study, NPs with proper particle size and zeta potential for intravitreal chemotherapy, low toxicity and high cell uptake were manufactured by adding CMD to TCs.^[42,44]^Further research is required to ensure the effectiveness and safety of TPH-CMD-TCs-NPs *in vivo*.

In conclusion, the TMC-cys conjugate was fabricated through covalent attachment of TMC with cysteine in the present study. Fabricated via self-assembly, the TCs-dextran NPs containing topotecan (TPH-CMD-TCs-NPs) had uniform particle size, spherical morphology, appropriate positive zeta potentials, and adequate topotecan EEs. The obtained results showed that NPs could efficiently deliver TPH into Y79 cells. Through thiolation of TMC, the advantages of TMC and thiomers for ocular delivery of drugs were combined, including permeation enhancing effects and muco-adhesion. The data obtained suggests a great potential for these self-assembled NPs as a promising medium for intravitreal drug delivery that offers an efficient technique for topotecan-assisted Rb chemotherapy.

##  Financial Support and Sponsorship

This study was funded by the research deputy of Tehran University of Medical Sciences (TUMS).

##  Conflicts of Interest

The authors report no conflicts of interest in this work.
